# A Multi-centre Survey of Acceptability of Newborn Screening for Sickle Cell Disease in Nigeria

**DOI:** 10.7759/cureus.2354

**Published:** 2018-03-21

**Authors:** Obiageli E Nnodu, Samuel A Adegoke, Osita U Ezenwosu, Ifeoma I Emodi, Ngozi I Ugwu, Chinatu N Ohiaeri, Biobele J Brown, John A Olaniyi, Hezekiah Isa, Chinedu C Okeke, Benard A Bene, Modupe T Balogun, Emmanuel C Okocha, John C Aneke, Juliana Olufunke J Lawson, Abjah M Usman, Ijeoma N Diaku-Akinwumi, Angela A Okolo, Yetunde T Israel-Aina, Mustapha Jamda, Oladapo W Aworanti, Frédéric B Piel, Adekunle D Adekile

**Affiliations:** 1 Department of Haematology & Blood Transfusion, University of Abuja, Abuja, NGA; 2 Department of Paediatrics, Obafemi Awolowo University Hospital, Ile-Ife, Osun State, NGA; 3 Department of Paediatrics, University of Nigeria Teaching Hospital, Enugu, NGA; 4 Department of Haematology & Blood Transfusion, Federal Teaching Hospital, Abakiliki, Ebonyi State, NGA; 5 Department of Paediatrics, Federal Medical Centre, Keffi, Nasarawa State, NGA; 6 Department of Paediatrics, University College Hospital, Ibadan, NGA; 7 Department of Haematology, University College Hospital, Ibadan, NGA; 8 Department of Haematology, Bingham University Teaching Hospital, Jos, Plateau State, NGA; 9 Non-Communicable Disease Division, Federal Ministry of Health, Abuja, NGA; 10 Department of Haematology & Blood Transfusion, Lagos State University Teaching Hospital, Ikeja, Lagos State, NGA; 11 Department of Haematology, Nnamdi Azikiwe University, Nnewi, NGA; 12 Department of Haematology, Nnamdi Azikiwe Teaching Hospital, Nnewi, NGA; 13 Department of Paediatrics, Zankli Medical Centre, Abujua, NGA; 14 Department of Haematology, University of Maiduguri Teaching Hospital, Maiduguri, NGA; 15 Department of Paediatrics, Lagos State University Teaching Hospital, Ikeja, Lagos State, NGA; 16 Department of Paediatrics, Delta State University Teaching Hospital, Asaba, NGA; 17 Department of Paediatrics, University of Benin, Benin City, Edo State, NGA; 18 Public Health & Tobacco Control, Community Medicine, University of Abuja, Abuja, NGA; 19 Small Area Health Statistical Unit, Mrc-Phe Centre for Environment & Health, School of Public Health, Faculty of Medicine, Imperial College London, London, GBR; 20 Department of Paediatrics, Kuwait University, Kuwait City, KWT

**Keywords:** new-born screening, sickle cell disease, acceptability, knowledge and attitude, genetic disease, nigeria, health survey, health care services, health care professionals, undergraduate students

## Abstract

Background

Sickle cell disease (SCD) is a major genetic disease that manifests early in life and may lead to significant morbidities. One of the health care services that have been effective in reducing the burden of SCD in developed countries is newborn screening (NBS) followed by pneumococcal vaccines, penicillin prophylaxis, and hydroxyurea treatment. Yet, in sub-Saharan African countries, where about 75% of annual affected babies worldwide are born, NBS programmes are largely unavailable. It is not clear whether this is due to technical challenges associated with setting up such programmes, or significant cultural and social barriers to its acceptance in such settings.

Objective

Our aim was to ascertain the attitudes to and acceptability of NBS in Nigeria among various socio-demographic groups including health professionals, undergraduate students, parents of children with SCD and SCD patients.

Methods

Data on socio-demographic characteristics, knowledge of SCD and attitude towards NBS were collected using a semi-structured pre-tested questionnaire from April to July 2014 across 15 health institutions and university campuses in Nigeria. Data were collected from 1,301 respondents across Nigeria.

Results

There was good knowledge of SCD as an inherited blood disorder. Although 86% of respondents (n = 1,119) supported NBS, there was a statistically significant relationship between support for NBS and age (p = 003), educational status (p = 000) and religion (p = 000).

Conclusion

This study suggests that there is a good acceptability of NBS across Nigeria. The main barriers to its use are likely to be financial and practical, rather than social or cultural.

## Introduction

Sickle cell disease (SCD) is a major genetic disease that manifests early in life and may lead to a lifelong illness with complications affecting almost every organ system in the body. The prevalence of sickle haemoglobin is between 20 and 30% in countries like Nigeria [1], Cameroon, the Democratic Republic of the Congo, Ghana and up to 45% in parts of Uganda. According to the World Health Organisation (WHO), the majority of children with the most severe form of the disease die before the age of 5 years, usually from an infection or severe blood loss. Therefore, the 63rd World Health Assembly, held in May 2011, adopted a resolution [2,3] calling upon affected countries to strengthen their response to SCD by increasing awareness about the global burden of haemoglobin disorders, promoting equitable access to health services and providing technical support for the prevention and management of the diseases. One program that has been effective in reducing the burden of SCD in developed countries, particularly in the US is newborn screening (NBS) [4,5]. So far, only a few African countries like Ghana, Benin, the Democratic Republic of the Congo and Uganda have offered NBS as pilot projects with external funding, but none has yet managed to scale up to a nation-wide effective screening program [6-10].

Although SCD is a major non-communicable disease (NCD) in Nigeria, with 2% prevalence in infancy [11], little attention has been paid to its prevention and management until recently. In order to address Nigeria’s 90,000+ annual births affected by sickle cell anaemia and the childhood under-five mortality associated with it, the government established six Millennium Development Goals (MDG) sickle cell centres between 2012 and 2013, one in each of the geopolitical zones of the country. Each centre was equipped with one BioRad VARIANT^TM^ high-performance liquid chromatography (HPLC) machine. The centres were meant to set up NBS and outreach services within the local communities aimed at early detection of individuals affected with SCD, health maintenance and comprehensive care as well as counselling and education of those with sickle cell trait. Almost six years later, the NBS program for SCD has been slow to take off and only the centre in North Central Nigeria has started operation at low capacity.

The acceptability of NBS for most of the country is unknown. Due to previous reluctance of target populations to accept health care services which are meant to benefit them, as was the case with immunization services [12], the Sickle Cell Support Society of Nigeria (SCSSN), which is the umbrella body for experts, patients, NGOs and others with interest in SCD in Nigeria and the Diaspora, conducted an exploratory survey to investigate the knowledge and attitude of different segments of the Nigerian population about SCD and towards different strategies for early diagnosis and reduction in the prevalence and burden of the disease in the country. The specific objectives were to ascertain the knowledge and attitudes of health professionals, parents, undergraduates and SCD patients towards NBS and its acceptability among the study population.

## Materials and methods

Study design

This was a multi-centre, cross-sectional assessment of the knowledge and attitude of health professionals, parents of SCD patients, individuals with SCD and undergraduate students towards NBS. A copy of the questionnaire is shown in Appendix 1 to illustrate the choices of answers available.

A multi-stage sampling technique was used to select a mixture of public-private Sickle Cell and Clinical Centres (i.e., newborn screening centres and tertiary health care and other centres affiliated to the SCSSN), in each of the six geopolitical zones of the country. In each centre, individuals with SCD, parents of SCD patients, health care workers, were selected through simple random sampling as they arrive in the clinics. The undergraduate students of the universities affiliated to these centres were selected by cluster sampling technique.

All the categories of respondents were administered the pretested data collection tool. The data collection tool is a semi-structured questionnaire adapted from Wonkam et al. [13]. The questionnaire written in English was designed to collect information from respondents on socio-demographic characteristics, knowledge about SCD, attitudes towards SCD screening policies and SCD-related diagnosis. Data were collected using this questionnaire between April and July 2014 across 15 health institutions and university campuses in Nigeria.

“Knowledge” in this study refers to what the respondents know about the cause of SCD, either as a disease entity inherited from parents, transmitted through contact with SCD patients, or cultural/religious beliefs (e.g., curse/punishments from God, or a dead child that reincarnated in the family).

“Attitude” in the context of this study refers to the perception of the participants, whether NBS should be carried out and, if so, at what time: before marriage, during pregnancy, after the birth of newborn babies or if family members of SCD patients should be screened.

Ethical issues

Ethical clearance was obtained from the Health Research Ethics Committee of University of Abuja Teaching Hospital (FCT/UATH/HREC/PR/352) and validated in other research centres as required. The aims and objectives of the study were explained to the respondents verbally. A written consent was obtained from each participant before enrolment in the survey. All identifiers of the respondents were removed from the data collection tool to encourage the participants to give honest responses to them.

Data analysis

Data collected from all the participating centres were manually entered into a Microsoft Excel database and checked for errors. Socio-demographic characteristics were presented in frequencies and percentages. Awareness of each component of knowledge (e.g., causation, possibility to diagnose in utero, soon after birth and any other time) and attitude towards screening for SCD (including before marriage, during pregnancy, after birth and reasons for each decision; practice of screening for parents of SCD clients) were presented in frequencies and then later cross-tabulated against age, sex, educational status, religion, income, marital status and number of living children. Statistical significance between different groups was established using the Chi-square test with a level of significance set at p < 0.05. The Likert package in R 3.4.3 was used to visualize the survey results.

## Results

There were 1,301 respondents. 51.6% (671) of whom were males and 46% (604) females. Twenty-six respondents (2%) did not indicate their gender. The mean age was 29 years, age range (19–49) years. The survey included 151 SCD patients (11.6%), 132 parents of individuals with SCD (10.2%), 627 undergraduate students (48.2%), and 24 members of the SCSSN (1.8%) as described in Table 1.

**Table 1:** Socio-demographic characteristics of respondents (n = 1,301).

	n (%)						
Variable			Female	Male	Missing	Total	
Age Group		<21	84 (55.6)	66 (43.1)	1 (0.7)	151 (100)	
		21-30	331 (42)	447 (56.7)	10 (1.3)	778 (100)	
		31-40	104 (51.7)	96 (47.8)	1 (.5)	201 (100)	
		41-60	55 (66.3)	24 (28.9)	4 (4.8)	83 (100)	
		61+	18 (46.2)	19 (48.7)	2 (5.1)	39 (100)	
Marital Status		Missing	12 (30.8)	19 (48.7)	8 (20.5)	39 (100)	
		Single	382 (41.0)	537 (57.5)	15 (1.6)	934 (100)	
		Married	206 (61.7)	120 (35.9)	8 (24)	334 (100)	
		Separated	10 (58.8)	7 (41.2)	0	17 (100)	
		Missing	6 (37.5)	7 (43.7)	3 (18.7)	16 (100)	
Religion		Roman Catholic	147 (49.8)	140 (47.5)	8 (2.7)	295 (100)	
		Protestant	129 (45.9)	146 (52)	6 (2.1)	281 (100)	
		Pentecostal	191 (45.11)	230 (54.3)	3 (0.7)	424 (100)	
		Muslim	105 (46.5)	117 (51.8)	4 (1.7)	226 (100)	
		Traditional	15 (46.8)	16 (50)	1 (3.1)	32 (100)	
		Missing	17 (93.5)	22 (51.2)	4 (9.3)	43 (100)	
Level of Education		No Education	10 (66.6)	5 (33.3)	0	15 (100)	
		Adult/Qur’anic/Primary	11 (84.6)	2 (15.4)	0	13 (100)	
		Secondary	111 (53.4)	94 (45.2)	3	208 (100)	
		Tertiary	460 (44.4)	555 (53.6)	20	1035 (100)	
		Missing	12 (40)	15 (50)	3 (10)	30 (100)	

**Figure 1:**
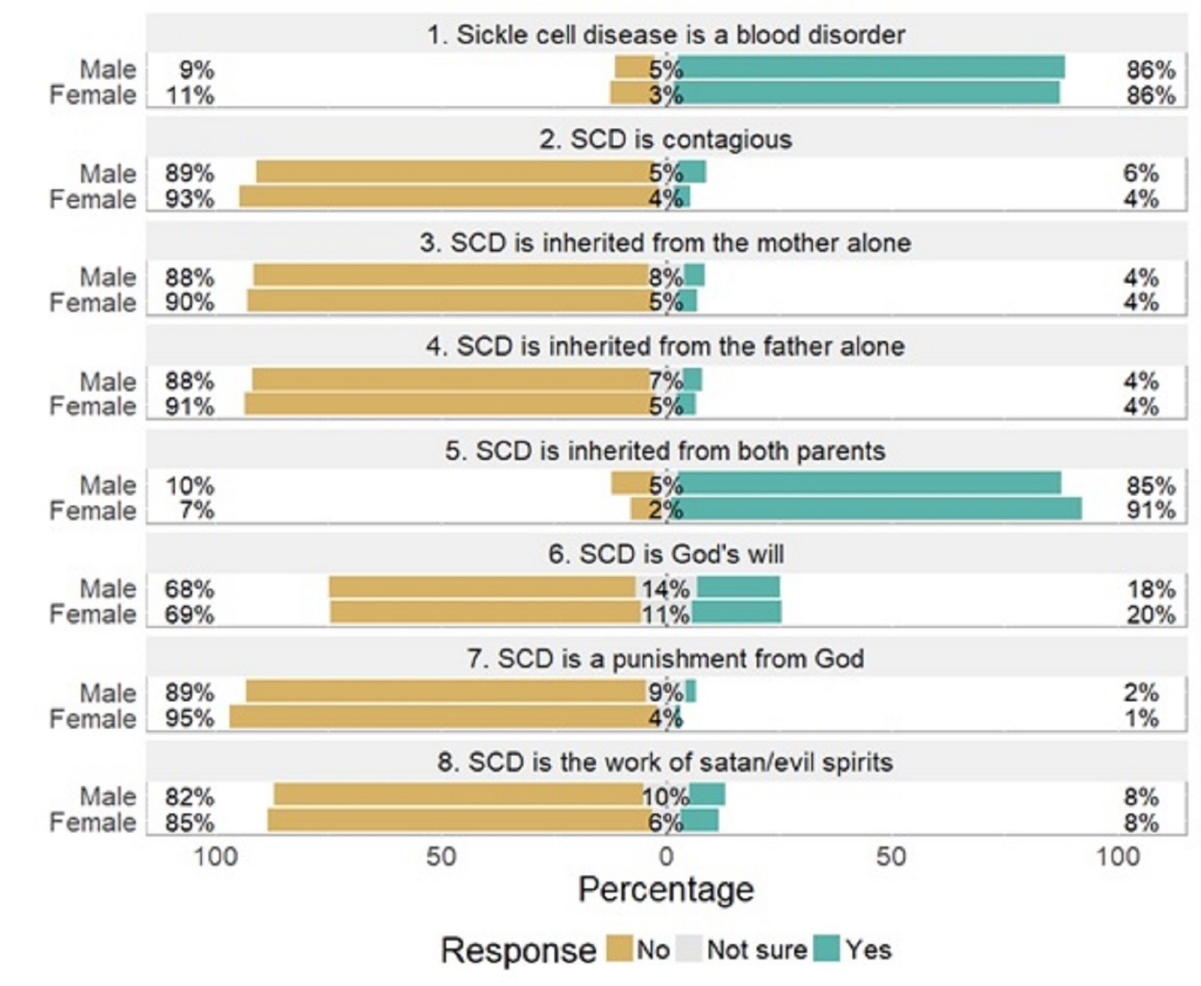
Knowledge of sickle cell disease among survey respondents by gender. SCD: Sickle cell disease.

Overall, there was good knowledge of SCD as an inherited blood disorder (79.4%). 84.3% of respondents were aware that the disease could be inherited from both parents. Forty-six individuals (3.5%) were aware of and disclosed their sickle cell trait status as AS/AC. 73.4% of respondents were aware of NBS. 67.3% were aware that SCD can be diagnosed before birth (prenatal diagnosis) and 84.0% believed that SCD can be diagnosed at any time in the life of a person or during pregnancy. Figure 1 illustrates both the knowledge of SCD among survey respondents and significant differences in the knowledge of SCD by gender. 86.1% of respondents conceptually supported NBS. The form of SCD screening considered most acceptable was before marriage (95.7%) while the least acceptable was prenatal diagnosis (73.8%). The screening of family members and all pregnant women was also considered acceptable by 84% and 81.3%, respectively.

A total of 1,119 (86.1%) respondents supported NBS. The most acceptable form of SCD screening was that before marriage (1,246, i.e. 95.7%) while the least acceptable was prenatal diagnosis (961, i.e. 73.8%). The screening of family members and all pregnant women was also acceptable by 1,098 (84%) and 1,057 (81.3%), respectively.

**Figure 2:**
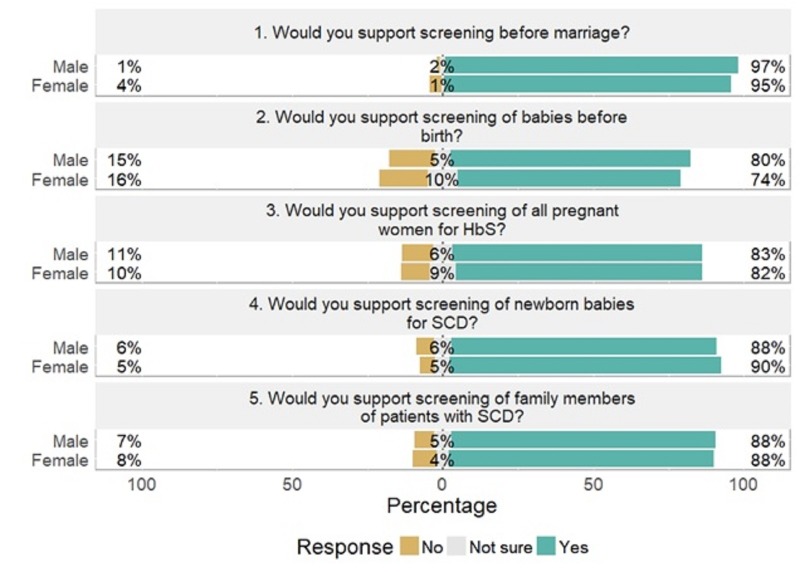
Attitudes towards genetic screening among survey respondents by gender. SCD: Sickle cell disease.

Cross-tabulating the support for NBS against gender, 90% of females supported NBS against 88% of male respondents (Figure 2). NBS was supported by at least 79% of respondents in all age groups, with a maximum acceptability in those aged 21-30 (90.6%). There was a statistically significant difference in support for NBS among those with tertiary education, secondary and adult/Qur’anic education (p = 014). For marital status, 808 (89.6%) of respondents that were single, 293 (88%) of married and 12 (76%) of those separated/widowed supported NBS. Regarding religion, 271 (92%) of Roman Catholics, 642 (91%) of protestants/Pentecostals, 78% of Muslims and 85% of traditional religion adherents supported NBS. These findings attest to the achievements of the government and various interest groups which have been carrying out advocacy and community mobilization activities to raise awareness about SCD and its prevention. However, this level of support requires being matched with action through bringing the screening closer home to the newborns in various communities.

**Figure 3:**
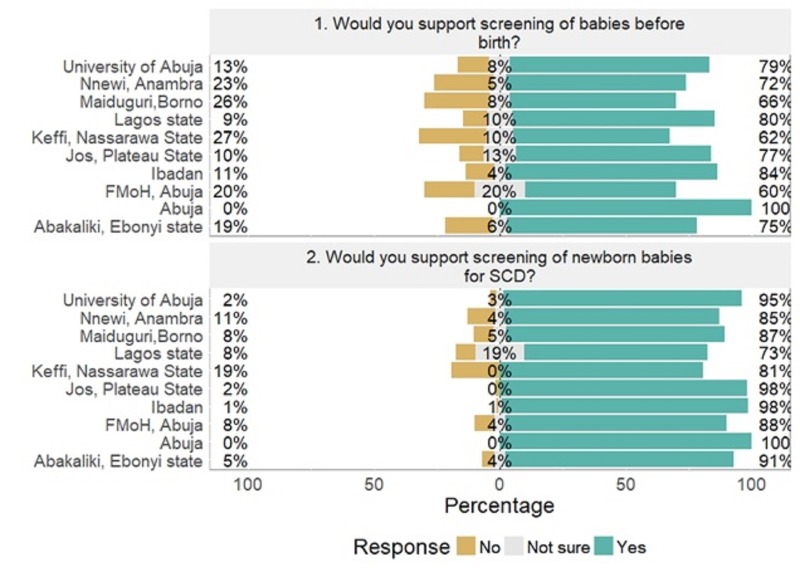
Differences between the regional centres included in the survey. SCD: Sickle cell disease.

Regional differences in support for NBS are shown in Figure 3. There was a statistically significant relationship between support for NBS and age, educational status, religion and category of respondents. Health care providers and individuals with SCD had the highest support for NBS (p = 0.000). There was a statistically significant difference (p = 0.079) between acceptability among the young (21-30 years old) and the eldest (≥60 year old). More singles supported NBS compared to married respondents although it was not significant (p = 0.059). Similar levels of acceptance among educational levels were observed but there was a statistically significant difference between educated respondents and those with no education (p = 0.001). Muslims reported lowest levels of acceptance when compared with Pentecostals, and other religious groups (p = 0.01).

There was a statistically significant relationship between support for NBS and age, educational status, religion and category of respondents. Thus the variables that were statistically significant following bivariate analysis were tested using the multiple regressions model for significance. Health care providers and individuals with SCD had the highest support for NBS (p = 0.000). The younger age group, 20-21 year old, had the highest support for NBS while the least support was from persons aged 60 years and above (p = 0.079). More singles supported NBS compared to married respondents although it was not significant (p = 0.059). There were similar levels of acceptance among educational levels but there was a statistically significant difference between educated respondents and those with no education (p = 0.0014). Muslims reported lowest levels of acceptance when compared with Pentecostals, and other religious groups (p = 0.01). These are shown in Table 2.

**Table 2:** Relationship between support for NBS and selected socio-demographic characteristics. NBS: Newborn screening; NS: Not significant; SCD: Sickle cell disease.

Variable		Do Not Support	Support	Chi-square	p-Value	Remarks
Gender	Male	40	577	1:81	0.398	NS
	Female	33	519			
Age Group	<21	5	127			
	21-30	54	856			
	31-40					
	41-60	9	69	23.46	0.003	Significant
	>60	4	34			
Education	Nil			33.09	0.000	Significant
	Adult/Quranic	3	8			
	Secondary	14	174			
	Tertiary	54	897			
Religion	Muslim	18	173	35.91	0.000	Significant
	Pentecostal	20	379			
	Protestant	11	251			
	Catholics	19	253			
	Traditional	2	26			
Category	Health Care Worker	0	14	8.73	0.001	Significant
	Parent of SCD Child	11	113			
	Undergraduate Student	6	135			
	SCD Patient	33	554			

## Discussion

The key public health message from this study is the good acceptability of NBS (86.1%) across the majority of groups analysed. The Nigerian effort to establish universal newborn screening failed despite the huge investment by the government because the logistics of sample movement for NBS was not matched with funds, there was attrition of trained staff from the zonal centres which did not take ownership of the program by including equipment maintenance costs and consumables for the NBS in their recurrent expenditure. Therefore the reason for poor implementation of NBS on a national scale with full utilisation of installed equipment is not due to non-acceptance by the population but possibly due to the high cost of purchase of reagents to run the tests, which costs about $20 per test in a country where the minimum wage is about $60 per month. This may be applicable to other African countries which have relied on external funding for the various pilot NBS projects which have been executed so far [6,7,9,10,14]. The recent development of low-cost rapid point-of-care devices could potentially considerably reduce the costs of NBS in such settings. Additionally, the NBS centres are located in tertiary health care centres, while the majority of Nigerians access health care services at the primary and secondary services rather than the tertiary centres which have longer waiting times and higher cost.

Females and young adults were the most in support of NBS at 90.0% and 90.6%, respectively. More healthcare providers supported NBS compared to the uneducated. The greater, though non-significant support by young adults, is encouraging since they are in the reproductive age range, thus more likely to practice it. Muslims had the least support to NBS which is likely related to Islamic teaching. Though this was not addressed in the study, Islamic teaching supports acceptance of “genotype” since abortion for SCD is not acceptable. Thus there is a need to access adherents of Islam even before marriage, otherwise, it would be more challenging to intervene afterward. Currently there is some effort at mass mobilization and sensitization activities with advocacy to religious leaders to request for the “genotype“ of couples who intend to be joined in marriages, there is a poor uptake of premarital counselling among marriages conducted according to Islamic laws relative to Church weddings where premarital counselling and tests including haemoglobin “genotype” tests are pre-requisites to conducting weddings. As interest in premarital counselling and screening in this population grows, the quality and the source of procurement of “genotype” tests need to be substantiated among faithful adherents of both religious groups [15].

There is some knowledge gap about the possibility of newborn screening among parents with SCD children and in the other groups but once people became aware of it in question 17 of the survey instrument, they gave their support. Since NBS programs rely on public trust, this knowledge gap must be adequately addressed not only at all levels of healthcare but with a functional inter-sectoral collaboration, especially with the mass media. It is for this reason that the SCSSN has incorporated the training of program officers in media houses as part of their activities to increase awareness about SCD and to sensitize the public on the benefits of interventions such as NBS in our country. The newborn screening programme for Nigeria, designed as "spoke and hub" type in which the centres receive samples from the peripheral facilities within each geo-political zones for screening, was found to be inefficient as a result of sustaining the logistics involved. Although the integration of non-communicable disease care including SCD into the primary health care had been done by the government of Nigeria, such integration did not take into account newborn screening tests at that level perhaps due to the available technology for NBS then. The healthcare workers at primary health care were not sensitized on newborn screening as a means of improving child survival. However, with the advent of point-of-care screening tests, once health care workers at primary health care levels are trained, screening can be made routine for all newborns in Nigeria through integration into routine immunization services. This will remove the problem of logistics that have stalled the efficient screenings of newborns at the sickle cell centres. A model of evidence-based interventions for the control of SCD [16], which integrates NBS into existing public health systems such as immunization programs and the Midwives Services Scheme within the Primary Health Care System, has been articulated as a means of making the government sickle cell centres more operational.

A key advantage of this study is the large sample size of 1,301 respondents including various categories of respondents across a wide geopolitical and ethnic distribution in the country compared to more localised studies reported by other workers [13,17,18]. A key limitation of our study is that there might have been a bias in the respondents because some of them were attending a healthcare facility or were affiliated with higher educational institutions. The acceptability of NBS might be lower to remote populations or the poorest who would not attend such a facility.

## Conclusions

Majority of respondents (1,119; 86.1%) supported newborn screening. This is encouraging in view of the planned implementation of interventions at community level aimed at reducing the burden of SCD in Nigeria. However, the study also shows that more awareness needs to be created among those with less education and in some religious groups in order to increase the level of acceptance across all segments of the society.
